# Silver sulfide decorated carbonaceous sawdust/ES-PANI composites as salt-resistant solar steam generator

**DOI:** 10.1039/d2ra04362a

**Published:** 2022-10-11

**Authors:** Ahmed K. Sadoun, Ahmed Gebreil, Rania M. Eltabey, Doaa A. Kospa, Awad I. Ahmed, Amr Awad Ibrahim

**Affiliations:** Department of Chemistry, Faculty of Science, Mansoura University Al-Mansoura 35516 Egypt amr_awad@mans.edu.eg +20-1091313272; Nile Higher Institutes of Engineering and Technology El-Mansoura Egypt

## Abstract

Solar steam generation (SSG) is a potential approach for resolving the global water and energy crisis while causing the least amount of environmental damage. However, using adaptable photothermal absorbers with salt resistance through a simple, scalable, and cost-effective production approach is difficult. Herein, taking advantage of the ultra-fast water transportation in capillaries, and the large seawater storage capacity of wood, we develop a highly efficient natural evaporator. The wood wastes (sawdust) were carbonized at low temperatures to fabricate a green and low-cost carbonaceous porous material (CW). To enhance the salt resistance in high saline water, this evaporator was coated with polyaniline emeraldine salt (ES-PANI) which was synthesized through facile and cost-effective one-step oxidation of aniline. Furthermore, the composite was decorated with silver sulfide to increase the evaporation rate which reached up to 1.1 kg m^−2^ h^−1^ under 1 sun irradiation with 91.5% efficiency. Besides, the evaporator performs exceptionally well over 10 cycles due to the salt resistance capability of ES-PANI which generates a “Donnan exclusion” effect against cations in saline water. The Ag_2_S@PANI/CW evaporator may be a viable large-scale generator of drinking water due to its high efficiency for energy conversion, simple and low-cost fabrication approach, salt-resistance, and durability.

## Abstract 2

Solar steam generation (SSG) is a potential approach for resolving the global water and energy crisis while causing the least amount of environmental damage.

## Graphical abstract

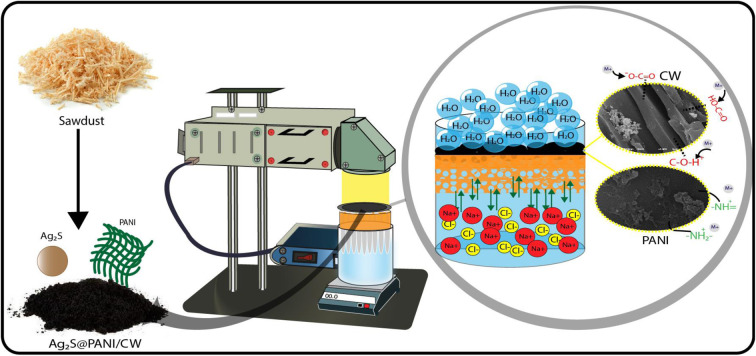

## 1. Introduction

Freshwater shortage and the global energy crisis have emerged as serious and frightening issues that must be addressed in our time. There are 1.4 billion cubic meters of water on our blue globe, but only 2.5% of it is freshwater, which is directly accessible to humans and animals. Approximately 70% of this small amount of freshwater is locked in glaciers and ice caps. The remaining 30% is ground and surface water, such as rivers, lakes, and marshes and its percentage has been steadily dropping due to frequent droughts and severe water pollution.^1^ In response, current technologies have been applied for water desalination such as reverse osmosis,^2^ electrodialysis,^3,4^ membrane distillation,^5,6^ and thermal distillation by heating bulk water.^7^ However, such technologies involve high energy consumption and increase greenhouse gas emission.

Recently, to meet the pressing need for freshwater at a minimal energy cost, the solar steam generation (SSG) technique is considered the best choice for the current issue as it involves a promising renewable green and cost-effective energy such as solar irradiation.^8,9^ However, because of thermal loss to the surrounding environment and bulk water, the traditional approach is inefficient, as a result, much effort has been devoted to improving SSG efficiency by reducing heat losses through the use of a bilayered structure. The bilayered device consists of a thermal insulation layer that floats over the surface of the water as a bottom layer and a photothermal absorber as a top layer.^10^ For the bottom layer must be low heat conductive materials that concentrate the heat at the surface and decrease the heat dissipation to the bulk. Wood,^11^ polyurethane foam (PU)^12^ as well as polystyrene foam (PS)^13^ have recently been employed as insulators in the fabrication of high-evaporation-efficiency SSG devices. Additionally, the top layer must have the capability to convert the sunlight to thermal energy effectively and localize the heat at the air/water interface and therefore, accelerating the water evaporation.

The interfacial solar steam production system depends significantly on the properties of the photothermal material. To successfully convert solar energy to heat, the evaporator must meet several characteristics, including strong solar energy absorption across the spectrum, minimal heat loss, durability, and low-cost synthesis. Carbon-based materials,^14^ plasmonic metal nanoparticles,^15^ metallic oxides,^16^ metal sulfides,^17^ and organic polymers^18^ have all been used as evaporators for SSG performance during the last several years. Carbonaceous materials including graphite,^19^ carbon black,^20^ carbon nanotubes,^21^ graphene (GO),^22^ hollow carbon spheres,^23^ and reduced graphene oxide (rGO)^24^ have drawn a lot of attention because of their excellent photothermal conversion efficiency and chemical stability. Moreover, carbon-based materials obtained from natural biomasses are excellent prospects for photothermal applications. Due to their economic potential, biomass resources such as wood, sawdust, straw, and plastic wastes are increasingly employed as a prospective source of energy.^25^ The carbonization process has evolved into a robust and versatile technology for synthesizing carbon compounds from organic biomass or biochar, thanks to a high-temperature-induced pyrolysis mechanism.^26^ Many researchers were impressed by this strategy and efficiently constructed different photothermal absorbers using common and low-cost organic porous materials such as foam,^27^ lotus seedpods,^28^ wood,^29^ bamboo,^30^ and cotton.^31^ Wood is one of the most abundant cellulosic biomasses with low-tortuosity, hierarchical, and mesoporous structures. High amounts of wood sawdust are produced as wastes in many industries and its disposal by burning increased the environment pollution. Hence, the recycling of sawdust wastes by the conversion to a corbon-based material is a promising strategy to decrease the pollution and fabricate cost-effective evaporators.

One of the promising photothermal materials is metal sulfide-based semiconductor materials (MSSMs) due to their distinct internal structures, strong optical absorption, water supply pathways, and the requisite solar conversion capacity.^17^ Silver sulfide (Ag_2_S), which has a tiny bandgap of (0.9 to 1.05 eV),^32^ is a potential selective photothermal absorber among all semiconductors. Furthermore, it exhibits unique features such as light absorption, antibacterial capabilities, high stability, and photoluminescence, as well as outstanding near-infrared light response.^33,34^ Hence, the incorporation of Ag_2_S into the carbonized wood enhances the solar absorptivity of the evaporator and thus increases the evaporation rates. On the other hand, the brine evaporates quickly during the evaporation process, leaving salts on the surface of the photothermal material. The salt crystals would considerably block water supply pathways, reduce vapor pressure, obstruct sun absorption, and lower evaporation efficiency dramatically over time.^35^ Various new photothermal evaporators have been modified to boost the effectiveness of the salt resistance to meet these difficulties.^36–38^ For effective solar desalination performance, new techniques for fabricating porous ionic polymers (PIPs) as salt-rejection photothermal absorbers have been devised.^39,40^ The porous structure of PIPs, which acts as a channel for water evaporation, and its ionic framework, which acts as a barrier for salt ions that prevents them from accumulation due to the “Donnan repulsion” phenomenon,^41^ promote the usage of these materials in this process. Among the porous ionic polymers, due to their unique attributes, including as low cost, ease of fabrication, capability to convert light to heat, excellent capabilities of adhesion, and hydrophilic properties, polyaniline (PANI) is the best choice.^42^ Emeraldine salt (ES), in particular, is considered the most efficient form of PANI in terms of sunlight absorption and resistance to salt accumulation during solar-driven water desalination.^43^ Overall, developing solar absorbers with cost-effective and facile fabrication, salt resistance, and high steam generation properties is still challenging.

We find that natural plants possess substantial channels, which provide well-developed capillaries to enable a sufficient water supply to the surface of the evaporator. Among various biomass, wood possesses unique structures that are desirable for efficient photothermal applications. Hence, in this work, sawdust was recycled into carbon-based solar absorbers, and construct solar-driven interfacial steam generators to replace the high cost carbon-based absorbers. Moreover, we employed the use of carbonized sawdust as a green and low-cost carbonaceous porous material (CW) which was modified by silver sulfide to enhance the light absorption broadband. Furthermore, the ES-PANI was incorporated into the evaporator to improve the salt resistance in highly saline water. The simple, cost-effective, and scalable Ag_2_S@PANI/CW composite was fabricated as a highly salt-rejecting photothermal absorber in which CW increased the hydrophilicity of the membrane, Ag_2_S increased the heat localization on the surface, and ES-PANI enhanced the salt resistance through the “Donnan exclusion” effect. The Ag_2_S@PANI/CW can be tightly bound *via* polyvinyl alcohol (PVA) coating and assembled on the surface of the support (filter paper), thereby forming a stable composite membrane. The composite membrane was combined with PU which served as an insulating substrate to lower the heat loss to the bulk. PU is pierced and packed with cotton which is a hydrophilic substrate for cellulose capillary action to provide water to the surface. The physical and chemical properties of the Ag_2_S@PANI/CW evaporator were studied, as well as the performance of evaporation and desalination reusability. Under 1 sun irradiation, the Ag_2_S@PANI/CW composite has a great rate of evaporation of 1.1 kg m^−2^ h^−1^ and a solar-to-steam efficiency of 91.5%. This high value was achieved by combining a semiconductor (Ag_2_S), a carbonaceous material (CW), and a conjugated porous ionic polymer (ES-PANI), all of which had excellent broadband absorption efficiency. The implementation of the “Donnan exclusion” effect produced by the positive charges of the quinonoid and benzenoid structure of ES, besides electrostatic attraction of the functional groups of the CW against salt ions during the desalination process, would exhibit the excellent long-term performance of water evaporation with a high persistent effect of desalination for seawater over 10 cycles. The ion rejection of the solar steam generator may achieve 99.8% when used to treat heavy metal ions solution and real seawater. The fabricated composite exhibited validity in the desalination of seawater, purification of dye-contaminated water, and rejection of salts, and could pave the way to develop a high-production, cost-effective, and simple fabrication of photothermal materials for efficient SSG. Finally, this work can promote a promising strategy for developing cost-effective evaporators based on the recycling of biomass wastes, exhibiting superior scalability, reusability, and easy solar energy conversion advantages.

## 2. Experimental section

### 2.1. Materials

Without any further purification, all chemicals were used as received. Hydrochloric acid (HCl, 37%), silver nitrate (AgNO_3_), sodium sulfide (Na_2_S), ammonium persulfate (APS), aniline (99%), methylene blue (MB), and rhodamine B (RhB) were bought from Sigma-Aldrich chemicals. Sodium chloride (NaCl), methyl orange (MO), calcium nitrate (Ca(NO_3_)_2_·4H_2_O), potassium chloride (KCl), and magnesium sulfate (MgSO_4_·7H_2_O) were bought from Alfa Aeser. Sawdust was purchased from a carpenter shop.

### 2.2. Preparation of CW

**Fig. 1:**
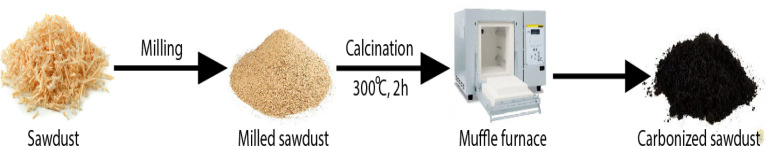
Schematic for sawdust calcination.

Sawdust was chopped to the size of 3 mm by a wood crusher. After that, it was washed with acetone and deionized water 3 times for eliminating any contaminants before being dried in the furnace at 70 °C for 8 hours. Subsequently, it was calcined at 300 °C for 2 h in a muffle furnace, with a heating rate of 5 °C min^−1^ then grounded and stored for further use as shown in Fig. 1.

### 2.3. Preparation of CW/PANI composite

PANI-ES was prepared by a process called chemical oxidative polymerization (COP) of aniline monomer using APS as a redox initiator in an acidic medium.^44,45^ To fabricate the CW/PANI composite, 1 g of CW was combined with 0.02 mmol of aniline dissolved in 70 mL of (1 M) hydrochloric solution, ultrasonically mixed for 30 minutes, and then magnetically stirred at 0 °C in an ice bath as a solution (A). 0.02 mmol APS was completely dissolved in 20 mL of (1 M) HCl aqueous as a solution (B). After that, solution (B) was added to solution (A), and the reaction was carried out at 0 °C for 2 hours until the solution turned dark green. The precipitate was centrifuged, washed with DI water and acetone 3 times, and dried in an oven for 6 h at 80 °C.

### 2.4. Preparation of Ag_2_S@CW/PANI composite

To synthesize Ag_2_S@CW/PANI composite, 1 g of as-synthesized CW/PANI was dispersed in DI water as a solution (A) followed by the addition of 0.0787 g of AgNO_3_ dissolved in 20 ml DI. After that, 5 g of sodium sulfide, was dissolved in 25 ml of deionized water and then added drop by drop to the mixture. Subsequently, as a complexing and stabilizing agent, 0.015 g of sodium citrate was dissolved in 10 mL of deionized water and added dropwise to the mixture. Then, the reaction was allowed to proceed for 12 h in the dark to avoid any impurity of metallic AgNPs. The solution was centrifuged, and the precipitate was washed three times with DI water and acetone. The precipitate was then dried in an oven at 80 °C for 6 hours.

### 2.5. Materials characterization

To characterize the as-synthesized samples, several analysis techniques were used. To demonstrate the existence of functional groups, FT-IR spectroscopy was performed using a MATTSON FT-IR-5000S spectrophotometer (spectral resolution 4 cm^−1^) with diamond attenuated total reflectance (DATR). X-ray diffraction (XRD) pattern measurements were collected using Rigaku (ULTIMA 4) diffractometer with a Cu K (*λ* = 0.154 nm) radiation source. In order to investigate the morphology and elemental composition of materials, SEM images, and EDX patterns, respectively, were taken using a scanning electron microscope (Joel JSM-6510). To identify and quantify all surface elements, the X-ray photoelectron spectroscopy (XPS) measurements were performed by K-Alpha (Thermo Scientific) spectrometer with Al Kα transition as the radiation source. The solar steam generation experiments were carried out on a SciSun-300 AAA solar simulator, with a power meter used to adjust the solar power. During the desalination process, the temperature of all thermal absorber surfaces was measured by thermocouple and their thermal images were taken with infrared radiation (IR) camera (htti-xintai). The ion concentrations of saline and desalinated water and dyes in solution were determined using inductively coupled plasma (ICP) mass spectrometry and UV-vis spectrophotometer, respectively.

### 2.6. Evaporation rate and efficiency test of SSG system under one sun irradiation

Solar steam production tests were carried out in a solar simulator as an irradiation source. A power meter was used to adjust the power density to 1 sun which is equal to 1 kW m^−2^. All SSG studies were performed for 1 h at a temperature of 22 °C ± 3 °C and a humidity of 50%, respectively. Every 5 minutes, the weight change of water due to evaporation was measured with an electronic analytical balance (accuracy: 0.1 mg). A thermocouple and an IR camera were used to capture the temperature distribution before and after illumination.

Eqn (1) was used to evaluate the evaporation rate (*v*):^43^1
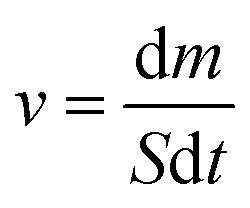
where *m* is the weight loss of water (kg), *S* is the photothermal membrane's surface area (m^2^), and *t* is the illumination duration.

The solar to steam efficiency (*η*) was computed *via*eqn (2):^12,43^2
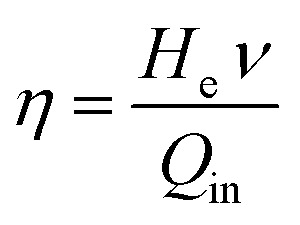
where *ν* (kg m^−2^ h^−1^) is the rate of vapor generation under steady-state conditions irradiation and *Q* in (mW cm^−2^) represents the incoming solar irradiation intensity. *H*_e_ is the sum of the latent heat and sensible heat enthalpy of the whole water-vapor phase shift, which is computed as follows:

3 *H*_e_ = *C*_p_Δ*T* + Δh

where, *C*_p_ is the specific heat capacity of water which is equal to 4.18 J g^−1^ k^−1^, Δ*T* is the change of temperature of the water, and Δh refers to the latent heat of vaporization on the relative temperature (2394 kJ kg ^−1^).

### 2.7. Bi-layer solar-driven water desalination device setup

Solar-driven water desalination bi-layer system involves two layers: PU foam, the bottom layer, which is 7 cm in diameter and 3 cm in depth and acts as a thermal insulator, and the top layer, which is the as-synthesized photothermal absorbers. The bottom layer was pierced and the holes were packed with cotton to transport water *via* capillary action to the top layer of the SSG device. The prepared absorbers (60 mg) were homogeneously dispersed in 7 mL of deionized water for 20 minutes. Then the absorber was placed into a 7 cm filter paper membrane and dried for 7 hours at 70 °C. Finally, the membrane was installed on polyurethane foam and the system was floated in 250 mL of saline water.

## 3. Results and discussion

### 3.1. SEM and EDX analysis

**Fig. 2:**
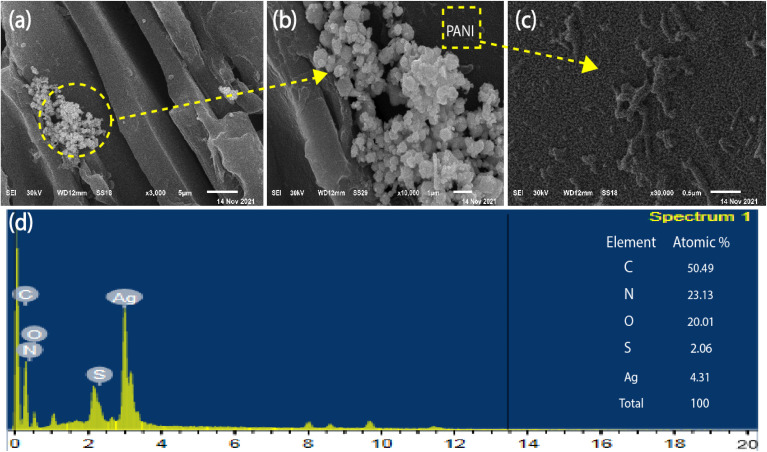
SEM images (a–c), and EDX (d) of Ag_2_S@PANI/CW.

Fig. 2 depicted the SEM image of the Ag_2_S@CW/PANI composite. We can see crevices formed by the carbonized wood as shown in Fig. 2a which can recover the lost energy of the reflected light, supplying an enlarged water/air interface for evaporation and forming an ideal substrate for the vapor to escape.^46^Fig. 2b showed that Ag_2_S particles successfully dispersed over the PANI/CW composite surface with a spherical structure.^47^ Granules-like structure of the PANI was shown in Fig. 2c which was homogenously distributed over the CW surface.^48,49^ On the other hand, EDX was also performed to determine the chemical composition of the prepared composite as well as the percentages of elements (Fig. 2d), which reveals the presence of carbon, oxygen, and nitrogen along with silver and sulfide indicating the successful preparation of the Ag_2_S@PANI/CW composite.

### 3.2. IR and XRD characterization

**Fig. 3:**
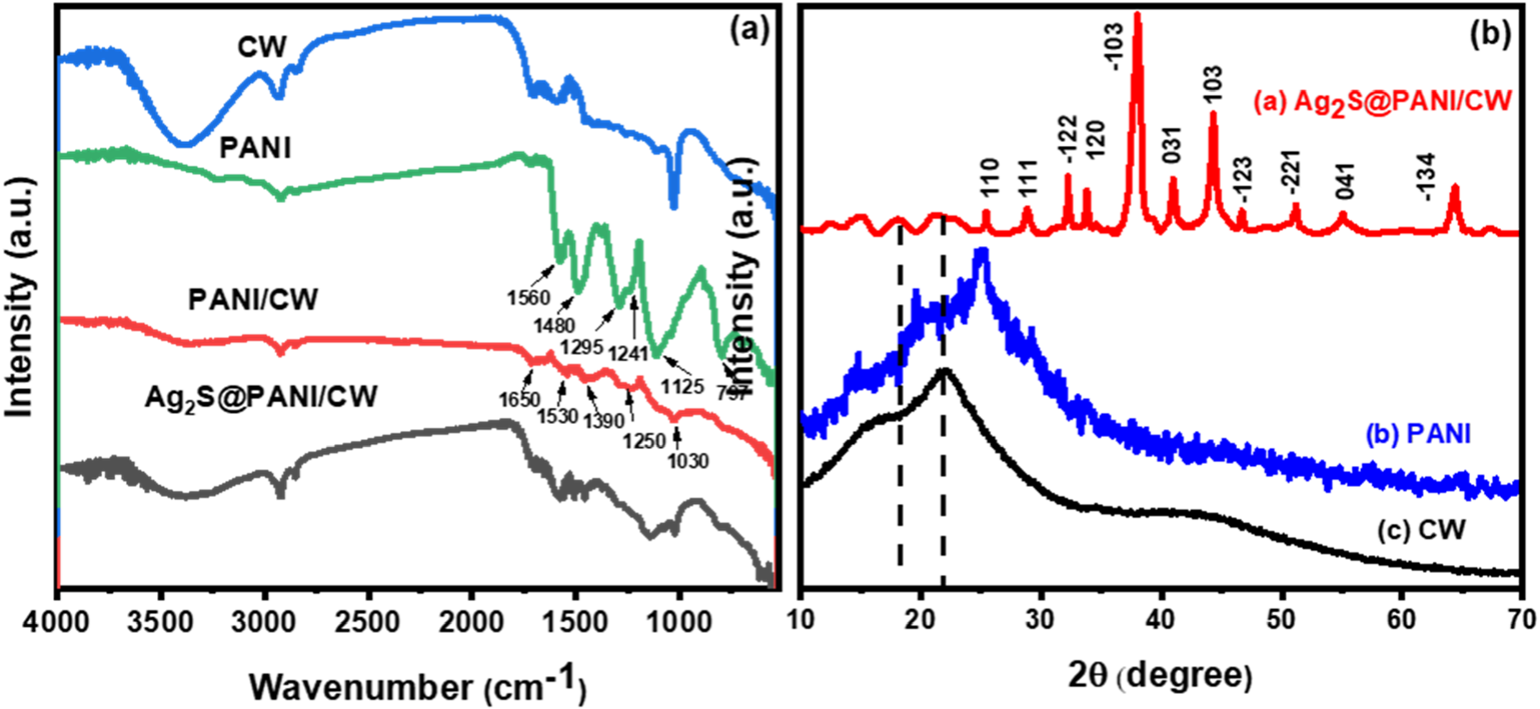
(a) IR spectra of Ag_2_S@PANI/CW, PANI/CW, PANI, and CW and (b) XRD pattern of Ag_2_S@PANI/CW, PANI, and CW, respectively.

The FTIR spectra of (CW, PANI, PANI/CW, and Ag_2_S@PANI/CW) were displayed in Fig. 3a. The FT-IR spectrum of CW showed several bands at 3381, 1734, 1596, and 1029 cm^−1^ correspond to strong hydrogen-bonded O–H, C=O, C=C, and C–O. While the band at 2931 and 2850 cm^−1^ represents C–C.^50,51^ The high intensity of the absorption band of phenolic and alcoholic hydroxyl groups is due to the lower pyrolysis temperature. For PANI, the peaks at ∼1560 and 1480 cm^−1^ correspond to the quinonoid and benzenoid rings C=C stretching, respectively.^43,52^ Meanwhile, the bands at ∼1295, 1241, and 1125 cm^−1^ are assigned to C–N and C–H bending of benzenoid and quinoid rings.^53^ The spectrum of PANI/CW showed the disappearance of some of the characteristic bands of the CW besides, it is almost the same as PANI, except they are smaller as a result of the attachment of poly aniline to the carbonized wood, which confirmed the successful coating of PANI over the carbonized wood.

To study the crystal phase structure, XRD was used to examine the crystal phase structure of CW, PANI, and Ag_2_S@PANI/CW (Fig. 3b). Carbonized wood exhibited just one wide diffraction peak at 22°, which may also be seen in Ag_2_S@PANI/CW XRD patterns with low intensity.^51^ In addition, three diffraction peaks were seen in pure PANI, corresponding to semi-crystalline planes (011), (020), and (200) which were found at 15.2°, 20°, and 25.3°, respectively.^54,55^ Furthermore, Ag_2_S@PANI/CW also revealed that the crystal plane and diffraction peaks of Ag_2_S were found at 25.3° (110), 28.9° (111), 32.3° (−112), 33.6° (120), 37.9° (−103), 40.7° (031), 44.4° (103), 46.7° (−123), 51.2° (−221), 54.8° (041), and 64.4° (−134).^56^ The monoclinic phase of silver sulfide is responsible for all of the observed crystal planes and diffraction peaks (JCPDS no. 14e0072) which confirmed the successful loading of silver sulfide on the PANI/CW composite.^57^

### 3.3. XPS analysis

**Fig. 4:**
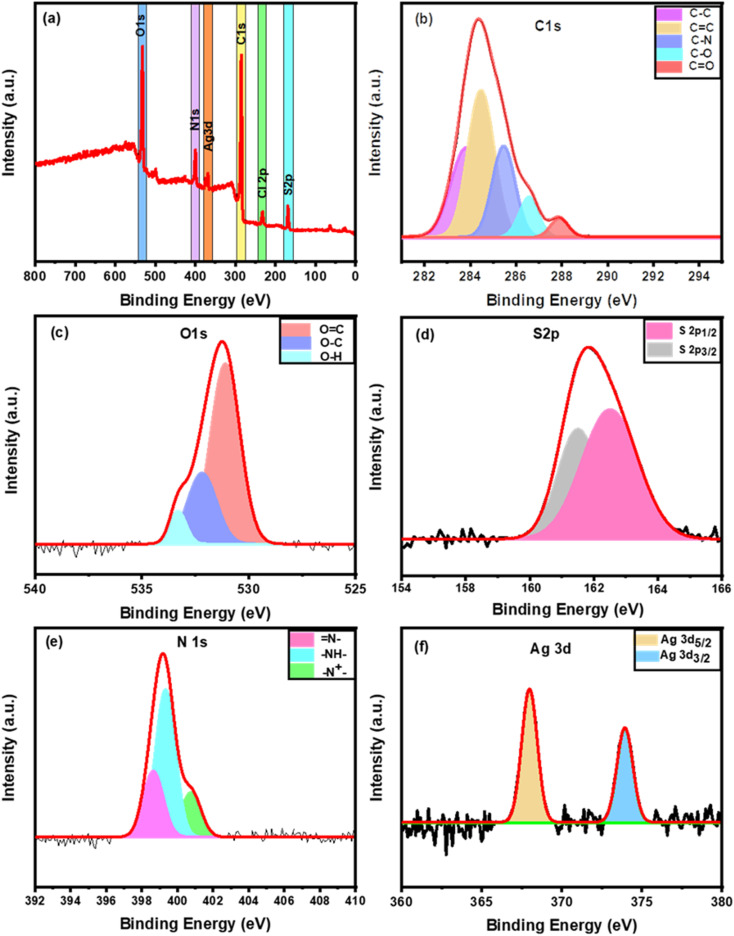
XPS of Ag_2_S@PANI/CW (a) XPS total survey spectra, and (b–f) XPS spectra of C 1s, O 1s, S 2p, N 1s, and Ag 3d, respectively.

The surface chemical composition and chemical state of the Ag_2_S@PANI/CW were investigated using XPS analysis, and the findings were shown in Fig. 4. Fig. 4a depicted the results of the XPS survey as a whole. The peaks located at 532.36, 400.12, 285.4, 168.9, 220, and 368.9 eV were assigned to binding energies of oxygen (O 1s), nitrogen (N 1s), carbon (C 1s), sulfur (S 2p), chloride (Cl 2p), and silver (Ag 3d), respectively. As shown in Fig. 4b, the XPS spectra of C 1s were separated into five Gaussian peaks that correspond to carbon atoms in various functional groups. The carbon in C–O bonds emerges at 283.64 eV, whereas in the carbonyl group (C=O) at 288.28 eV. Furthermore, the C in C=C bonds is at 284.2 eV, and in C–N at 285.27 eV.^58–60^ The XPS spectra of oxygen were divided into three peaks (Fig. 4c), at 531.2, 532.5, and 533.49 eV, which correspond to the O=C, O–C, and O–H bonds, respectively.^60^ The binding energies of S2p_3/2_ and S2p_1/2_ are 161.5 and 162.7 eV, respectively (Fig. 4d).^61^ As shown in Fig. 4e, the N 1s spectrum, has 3 peaks at binding energies of 398.49, 399.26, and 400.98 eV, respectively, corresponding to benzenoid amine bonds (=N–), quinoid imine bonds (–NH–), and nitrogen cationic radicals (N^+^) of PANI, indicating that the emeraldine salt of PANI was successfully formed as well as the presence of Cl^−^ in the total survey.^43,62^ Furthermore, the high-resolution spectra of Ag 3d were shown in Fig. 4f. Ag 3d_3/2_ corresponds to the high binding energy of 374.1 eV, while Ag 3d_5/2_ corresponds to the low binding energy of 367.5 eV which corresponded to the Ag^+^ cations in the Ag_2_S.^63^

### 3.4. SSG performance

**Fig. 5:**
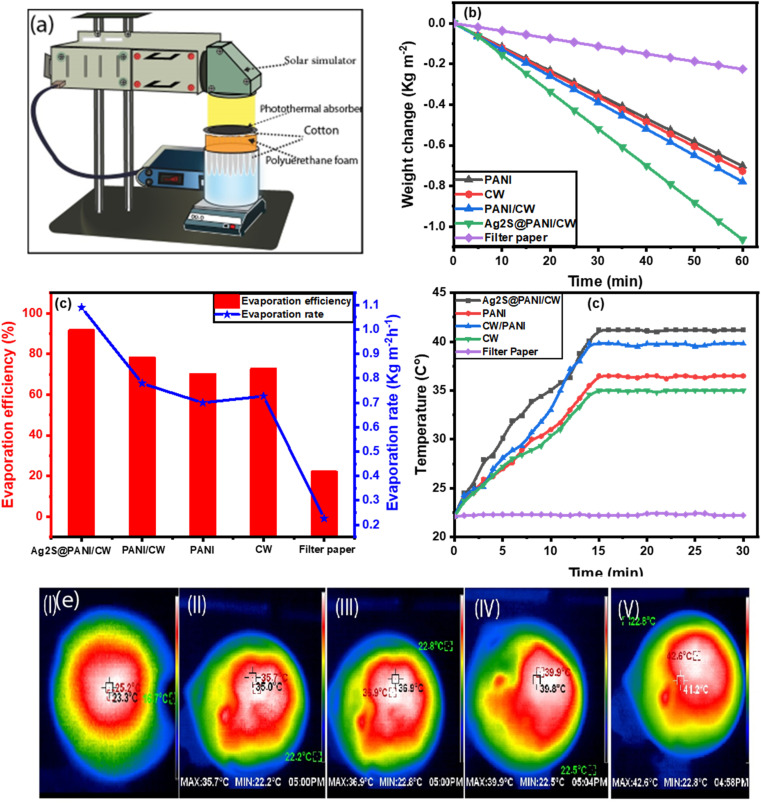
(a) Schematic of SSG system, (b) water mass loss of different solar absorbers, (c) evaporation rate and efficiency of evaporators, (d) temperature change at the evaporator surface after 30 min under 1 solar irradiation, and (e) IR thermal images of filter paper (I), CW (II), PANI (III), PANI/CW (IV) and Ag_2_S@PANI/CW (V) after 30 min under 1 solar irradiation, respectively.

For evaluation of water evaporation performance for the different synthesized photothermal absorbers; CW, PANI, PANI/CW, and Ag_2_S@PANI/CW, SSGD device was designed and applied as illustrated in Fig. 5a. Briefly, the photothermal absorbers collect incident sunlight from all directions and develop a heat localization area where steam is generated. Filter paper, being a porous hydrophilic substance, acts as a small reservoir, supplying water molecules to the photothermal layer continually. Whereas, polyurethane foam is chosen as a thermally insulating substrate to prevent dissipation of heat to the bulk. Under solar light irradiation, the mass change of DI water was measured by an electronic scale for 1 hour under 1 sun irradiation. At appropriate periods, which are 5 minutes of irradiation, the cumulative time-dependent mass variations of the water of filter paper and photothermal evaporators were accounted as shown in Fig. 5b. The figure showed that the water mass changes of filter paper, CW, PANI, PANI/CW, and Ag_2_S@PANI/CW absorbers were 0.226, 0.728, 0.701, 0.779, 1.09 kg m^−2^, respectively. As can be observed from the data, the mass loss for Ag_2_S@PANI/CW is substantially higher than those of other thermal absorbers, owing to the strong absorption coefficient of the carbon as well as the incorporated silver sulfide. The small band gap (0.9 to 1.05 eV) of Ag_2_S,^32^ increases the solar-to-heat conversion of the membrane due to the fast recombination of h^+^/e^−^ species and thus increases the evaporation rate. Because of its porous nature, CW has good optical properties, improving multi-scattering-induced light trapping, lowering light transmittance, and lowering light reflection to the surrounding.^51^ Furthermore, PANI provides even more value to the composite due to its unique photothermal conversion capacity, high adhesive qualities, and good hydrophilicity. In addition, the performance of solar evaporation was evaluated by determining the evaporation rate and evaporation efficiency under one solar illumination (Fig. 5c). It was observed that the Ag_2_S@PANI/CW nanocomposite exhibited a great evaporation rate and efficiency achieved up to 1.09 kg m^−2^ h^−1^ and 91.5%, respectively.

Moreover, the surface temperature of the photoabsorbers is plotted in Fig. 5d as a function of the irradiation time by a thermocouple sensor probe throughout the vapor production process. The average surface temperature of absorbers increased rapidly from 23.3 °C for filter paper reaching 36.9, 35, 39.8, and 41.2 °C for PANI, CW, PANI/CW, and Ag_2_S@PANI/CW, respectively, in about 15 minutes. The Ag_2_S@PANI/CW absorber had the greatest temperature after 15 minutes, showing that solar energy was concentrated on this membrane's surface, resulting in the maximum evaporation rate. The infrared radiation (IR) camera was used to confirm the temperature rise of Ag_2_S@PANI/CW to further study heat localization performance under a light source of 1 kW m^−2^. Typical infrared photos of the temperature distribution of the photothermal absorber were taken every 30 min and were shown in Fig. 5e, which agreed with the obtained values in the temperature–time curve. In addition, the heat transfer and energy balance during the water desalination process were calculated using the following equations:^64,65^

The balance of the energy can be expressed as:

4 *Q*_Solar_ = *Q*_Evap_ + *Q*_Conduction_ + *Q*_Convection_ + *Q*_Radiation_

where *Q*_Solar_ represents the input of the solar energy = 1000 W m^−2^.

Conductive heat loss to the bulk (*Q*_Conduction_) can be calculated *via* the following equation:

5 *Q*_Conduction_ = *Ak*(*T*_1_ − *T*_2_)/Δ*l*

*A* is the cross-sectional area of the photoabsorber, *k* is the conductivity of the heat of water (0.599 W m^−1^ K^−1^), *T*_1_ and *T*_2_ represent the temperatures of two points in water bulk, whereas Δ*l* is the distance between the two points in the bulk (0.9 cm).

The convective heat loss (*Q*_Convection_) to the surrounding can be represented as:

6 *Q*_Convection_ = *Ah*(*T*_s_ − *T*_v_)

The convection heat transfer coefficient is expressed as *h* (10 W m^−2^ K^−1^), while *T*_s_ is the photoabsorber surface temperature (41.2 °C).

The radiative heat loss (*Q*_Radiation_) to the environment can be calculated using the equation:

7 *Q*_Radiation_ = *Aεσ*(*T*_s_^4^ − *T*_∞_^4^)

where *ε* is the emission of the evaporator (0.96), *σ* is the Stefan–Boltzmann constant (5.669 × 10^−8^ W m^−2^ K^−4^), *T*_∞_ is the temperature of the surrounding environment.

According to the above thermal study, only 0.0262% of the total energy loss in Ag_2_S@PANI/CW dissipated to the bulk water *via* top-down heat conduction. Furthermore, the convection energy loss was 1.53% of the heat; whereas the radiation loss to the surroundings was 7.03%. The effective energy of the evaporation is equal to 914.14 W m^−2^ which is corresponding to 91.4% of the total light input.

**Fig. 6:**
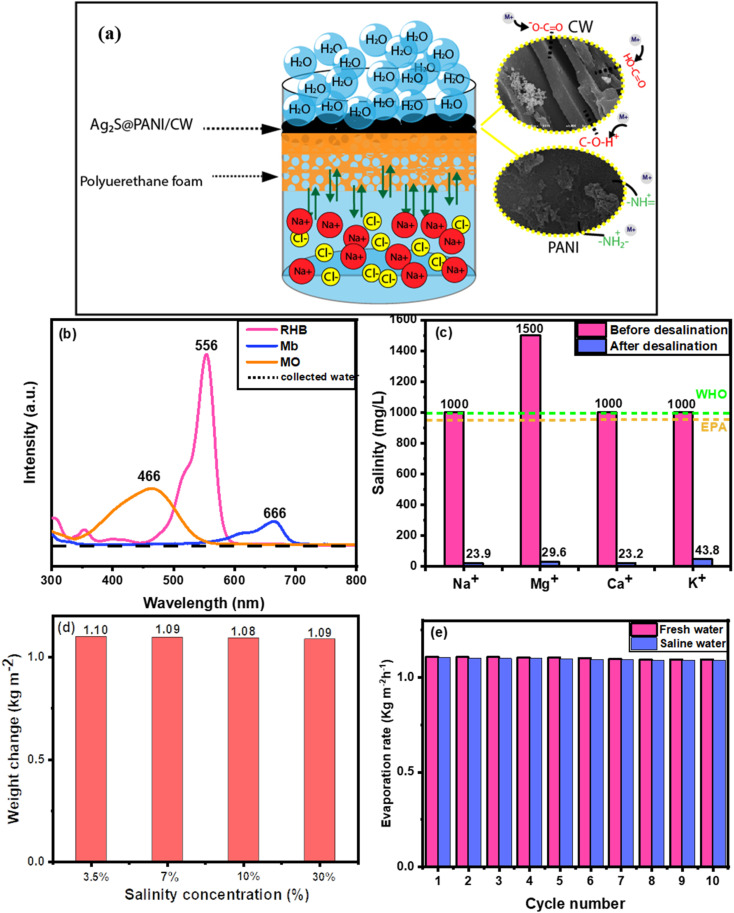
(a) schematic representation of the salt-resistant mechanism, (b) UV-vis absorption spectra of dye-contaminated water before and after desalination under 1 sun irradiation, (c) concentration of metal ions before and after desalination process, (d) water mass change of Ag_2_S@PANI/CW under different NaCl concentration, and (e) reusability test of the Ag_2_S@PANI/CW sample during 10 cycles under constant 1 sun irradiation for 60 min for each cycle.

The water quality was assessed after the desalination of artificial seawater using a condensing unit to collect the evaporated water. ICP-OES is used to determine the concentrations of Mg^2+^, Ca^2+^, Na^+^, and K^+^. The concentration of the four ions decreased significantly, as seen in Fig. 6c after desalination with the Ag_2_S@PANI/CW evaporator. The ion concentrations dropped dramatically from their original levels of 1500, 1000, 1000, and 1000 mg L^−1^, respectively, meeting the standard values of freshwater according to the world health organization (WHO) and Environmental Protection Agency (EPA) guidelines. The rejection potential of the Ag_2_S@PANI/CW was attributed to the functional groups of the CW sample, which act as a barrier against the seawater ions and capture them through electrostatic attraction as shown in Fig. 6a, as well as the repulsion between seawater ions and the cationic groups of (ES-PANI) framework. Fig. 6b showed that Ag_2_S@PANI/CW composite can be used to purify the polluted wastewater with 10 ppm of various dyes such as MO, RhB, and MB with great efficiency using solar energy. The absorption spectra of the contaminated wastewater before and after desalination displayed that the dyes have been completely removed from the evaporated water. Furthermore, to investigate the evaporation rate of the Ag_2_S@PANI/CW composite in the saline water, the SSG was performed with a series of different concentrations of NaCl (3.5%, 7%, 10%, 30%) under 1 sun illumination for 1 h which exhibited almost no difference in the water mass loss as displayed in Fig. 6d. The stability of the evaporation rates at different saline water is attributed to the salt rejection effect of PANI in the evaporator, hence, the (Ag_2_S@PANI/CW) composite can preserve the freshwater production capacity even in saline water.

In addition, ten cycles of evaporation tests in artificial saltwater (20 wt% saline) were done under 1 sun illumination for 1 hour for each cycle to perform the reusability test of Ag_2_S@PANI/CW absorber in solar desalination of seawater. Due to the presence of polyaniline and function groups in the carbonized wood, which has the potential to reject salt ions, efficiencies virtually did not decline by cycles in the case of saline water.

## 4. Conclusion

In summary, we have successfully fabricated a solar-driven water desalination system based on carbonaceous sawdust doped with Ag_2_S and paired with polyaniline to improve its salt rejection capability for highly effective solar-driven water desalination. The porous structure, low heat conductivity, and good chemical stability of the as-synthesized Ag_2_S@PANI/CW bilayer system, along with excellent light-absorbing, make it a promising SSG approach. The solar to steam conversion efficiency of the Ag_2_S@PANI/CW composite is up to 91.5% during one solar irradiation, and the rate of evaporation of 1.09 kg m^−2^ h^−1^ has been achieved. Due to the synergistic effect of the functional groups in the CW structure and polyaniline, the Ag_2_S@PANI/CW evaporator possesses efficient water delivery and high rejection of salts. As a result, for continuous evaporation of 10 hours under one solar irradiation, the evaporator showed stable evaporation. Also, a clear salt-rejection capability has been attained even in high saline water (20 wt%), implying long-term light to heat conversion capability for solar-driven water desalination. Furthermore, the successful removal of organic pollutants confirmed the ability of the membrane to produce safe drinking water. After analyzing most desalination experiments, the results revealed that the fabricated evaporator outperformed numerous earlier devices.

## Conflicts of interest

The authors declare no competing financial interest.

## Supplementary Material
